# Sarcopenia risk as a predictor of urinary incontinence in women aged 50 and older: a cross-sectional analysis

**DOI:** 10.1038/s41598-025-11963-2

**Published:** 2025-07-26

**Authors:** Wesam A. Debes, Munseef Sadaqa, Pongrác Ács, Kálmán Kovács, Viktória Prémusz, Márta Hock

**Affiliations:** 1 Doctoral School of Health Sciences, Faculty of Health Sciences, University of Pécs, Vorosmarty Street 4, Pécs, 7621 Hungary; 2 Physical Activity Research Group, Szentágothai Research Centre, Pécs, Hungary; 3 Institute of Physiotherapy and Sport Science, Faculty of Health Sciences, University of Pécs, Pécs, Hungary; 4 National Laboratory on Human Reproduction, University of Pécs, Pécs, Hungary; 5 HUN-REN–PTE Human Reproduction Research Group, Berne, Switzerland; 6 Department of Obstetrics and Gynaecology, University of Pécs, Pécs, Hungary

**Keywords:** Sarcopenia, Urinary incontinence, Muscle mass, Muscle strength, Women, Risk factors, Geriatrics, Urinary incontinence

## Abstract

Urinary Incontinence (UI) has multiple negative effects on women’s health. Multiple risk factors were investigated previously in different settings and populations. This study aims to determine the association between UI and the risk of sarcopenia and other associated risk factors among women above the age of 50 years in Hungary. This cross-sectional study included 215 women aged ≥ 50 years. Participants were recruited from community-dwelling and nursing homes. The survey included medical and obstetric information, UI was assessed using the International Consultation on Incontinence Questionnaire Lower Urinary Tract Symptoms Modules (ICIQ-FLUTS), and risk of sarcopenia was assessed by SARC-F. This study adhered to the Strengthening the Reporting of Observational Studies in Epidemiology Statement (STROBE) checklist for cross-sectional studies to report the results. The protocol for the study was registered on clinicaltrials.gov under the identifier NCT05313360. Four hundred and thirty-four individuals were invited to participate, and at the final stage, 215 participants were available for the final analysis. In addition to the risk of sarcopenia, age when giving the first delivery and the number of complications during birth were risk factors for UI. Further longitudinal studies are recommended to investigate the association over extended periods.

## Introduction

Urinary incontinence (UI) can be defined as “any complaint of involuntary loss of urine”^1^among women, it’s usually related to bladder or pelvic floor muscles dysfunction^2^. UI is usually categorised into two primary subtypes: stress incontinence (SUI) and urgency incontinence (UUI). Stress incontinence refers to the complaint of urine leakage that is associated with coughing, sneezing or physical exertion. Urgency incontinence, on the other hand, is the complaint of urine leakage associated with a sudden desire for voiding^2^. All types of incontinence are more common among older age groups^3^. UI has a huge negative impact on the quality of life in addition to increased healthcare costs^4^.

Prevalence of UI had enormous variation in the literature, ranging from 5 to 70%, yet the majority of studies reported a prevalence of 25–45%^5^. Moreover, UI is found three times more prevalent among females in all age groups compared to males^6^. Additionally, the mean annual incidence of UI among women ranged from 1–9%^7^.

The data about the prevalence of UI is scarce in Hungary. One notable study conducted in 2001 found that 56% of them reported experiencing some sort of UI symptoms. Additionally, 36% of the participants identified themselves as having UI^8^. Another Hungarian study reported that 33.9% of the participants stated facing urinary leakage at some point in their lives. Also, 8.6% experienced UI multiple times per day, and only 1.0% reported continuous symptoms of UI^9^.

Multiple risk factors of UI have been studied in the literature. These factors can be classified into unmodifiable and modifiable^10^.

Sarcopenia is another important geriatric condition that is defined according to the European Working Group on Sarcopenia in Older People (EWGSOP) as” the presence of low muscle mass and low muscle function (low strength or low physical performance)”^11^. In 2018, the group updated the previous definition by considering low muscle strength as the primary measure for diagnosing sarcopenia^12^. Sarcopenia is usually accompanied by various consequences such as physical disability, falling, poor quality of life and sometimes a leading cause of death^13–15^. Due to its multiple negative health outcomes, sarcopenia is considered a main contributor to healthcare costs^16^.

The prevalence of sarcopenia varies across studies. Previous studies reported a prevalence ranging from 10 to 16% across various definitions used to define sarcopenia^17^. In Hungary, a recent study explored sarcopenia among 50-year-old or older women reported a prevalence of 31%^18^.

The pelvic floor is made mainly of the levator ani muscle group, the endopelvic fascia, and the supporting ligaments. Subsequently, weakness of these mechanisms can cause a failure of the urethra to produce enough pressure to resist the rise in intravesical pressure, causing incontinence^19^. Moreover, the abdominal muscles also have an important role in maintaining continence due to their synergistic and coordinated contribution to the function of pelvic floor muscles^20^.

Additionally, a previous study suggested that older adults who presented atrophy and weakness of the pelvic muscles experienced higher incidents of UI^21^. Furthermore, it has been suggested that individuals with UI who have undergone pelvic floor muscle training programs showed a significant improvement in incontinence symptoms^22^.

In light of the aforementioned information, sarcopenia might be a risk factor for UI as decreased pelvic floor and abdominal muscles strength is connected directly with the mechanism of UI. In addition, age is considered a risk factor for both sarcopenia and UI^23,24^. Also, the literature showed inconsistent findings about the association between UI and postmenopause, as some studies suggested that postmenopause was associated with UI, while others found UI occurred is linked to premenopausal and perimenopausal women^25^.

We noticed a lack of studies exploring the association between the risk of sarcopenia and UI among middle- and old-aged women. Hence, this study aims to investigate the association between risk of sarcopenia and UI in addition to demographic, health and obstetric risk factors among women above 50 years old in Hungary.

## Materials and methods

This cross-sectional study included 215 female participants in Hungary, using a non-probability convenience sampling technique. A survey developed by the researchers was utilised to collect data on demographics, medical history, and health status, in addition to ICIQ-FLUTS, and SARC-F. The target participants were community-dwelling as well as elderly home residents. The participants filled out paper-based surveys in the presence of qualified physiotherapists and nurses who served as data collectors, in addition, they facilitated the completion of the survey and answered any inquiries from the participants. The data collectors reviewed each survey to identify any missing data, accordingly, surveys with missing data were excluded from the analysis.

To reduce interviewer bias^26^ data collectors were unaware of the study hypothesis to prevent selective data collection consciously or unconsciously. While both the participants and the data collectors were informed about the general aim of the study, the hypothesis was deliberately not clearly stated.

We included women aged ≥ 50 years who agreed to fill out the survey. Participants were excluded if they were unable to provide reliable clinical information or understand the questions. In addition, individuals who had any neurological disorders (e.g., Parkinson’s, stroke, multiple sclerosis) or who lived outside of Hungary were excluded from the study. This study adhered to the Strengthening the Reporting of Observational Studies in Epidemiology Statement (STROBE) checklist for cross-sectional studies to report the results^27^.

Written informed consent was obtained from all study participants before their participation. The study adhered to the principles outlined in the declaration of Helsinki and received approval from the University of Pécs Clinical Centre Regional Research Ethics (Nr. 9080 - PTE 2021). The study protocol was registered on clinicaltrials.gov under the identifier NCT05313360.

### Measurement tools

Qualified participants answered questions about demographic data including age, height, weight, level of education, marital status, smoking status, number of diseases, medical history including previous surgeries, medication, and visits to the hospital in the last year. In addition, a history of urinary incontinence in the family was assessed.

Moreover, the survey included specific questions for females that comprise information about the complications during birth, in addition to the number and methods of deliveries (vaginal or caesarean). The duration of data collection was 16 months, from January 2022 – May 2023.

Body mass index (BMI) was calculated as weight (kg) divided by height (m^2^). Risk of sarcopenia was assessed by SARC-F, according to the European Working Group on Sarcopenia in Older People 2 (EWGSOP2) consensus, SARC-F was suggested as an appropriate tool to screen the risk of sarcopenia^12^.

The SARC-F questionnaire is composed of 5 self-reported questions, including the following outcomes: strength, assistance in walking, rise from a chair, climbing stairs, and falls. Each item is scored from 0 to 2. The cutoff point of ≥ 4 indicates a risk of sarcopenia; a higher result is interpreted as an increased risk of sarcopenia. In this study, we conducted the Hungarian-validated version of SARC-F^28^.

Lower urinary tract symptoms were assessed by the validated Hungarian version of the International Consultation on Incontinence Questionnaire-Female Lower Urinary Tract Symptoms Module ICIQ-FLUTS^29^ which was derived from the Bristol Female Lower Urinary Tract Symptoms (BFLUTS) questionnaire. ICIQ-FLUTS is considered an appropriate tool for screening urinary incontinence in research settings as it follows standard methods of psychometric testing, including content validity, construct validity, stability, and internal consistency. It investigates various lower urinary tract symptoms including filling, voiding and incontinence, in addition, the questionnaire evaluates the effect of these symptoms on the quality of life of individuals. The total score is 48, the subscales consist of filling symptoms scored from 0 to 16, voiding symptoms scored from 0 to 12, and incontinence symptoms scored from 0 to 20. A higher score of ICIQ-FLUTS indicates more severe lower urinary tract symptoms^30,31^.

### Statistical analysis

Descriptive statistics were presented as means and standard deviations for numerical variables, and percentages were used to express frequency. Normality of the data was assessed by the Kolmogorov-Smirnov test; consequently, due to the nature of our data, we used non-parametric tests, including Mann-Whitney U test to compare the difference between means of the nominal independent variables. Furthermore, to assess the correlation between incontinence symptoms and suggested risk factors, Spearman rank correlation was used. Multivariate linear regression to investigate the associations between incontinence symptoms (dependent variable) and the independent variables that were significant (*p* ≤ 0.05) in the univariate analyses, including history of incontinence in the family, age, risk of sarcopenia, age when giving birth in the first delivery, number of hospital visits during the past year, number of medications intake, number of diseases, and number of complications during delivery. The regression results are presented as standardised coefficients (β), with respective 95% confidence intervals (CIs). The significance level for all tests was established at *p* < 0.05. Statistical analyses were carried out using IBM SPSS Statistics for Windows, version 27.0 (IBM SPSS, Armonk, NY, USA: IBM Corp.)

An a priori sample size calculation was done using G*Power software (version 3.1.9.7; Heinrich-Heine-Universität Düsseldorf, Düsseldorf, Germany). With a correlation coefficient of 0.30, a significance level of 0.05 and a statistical power of 0.95^33^. Based on these assumptions, 115 women were needed. Thus, 215 participants were adequate to run the analysis.

## Results

**Fig. 1:**
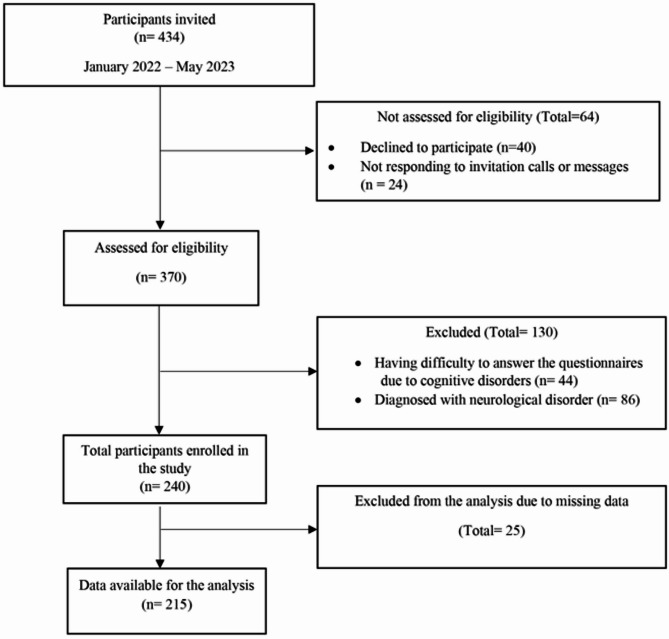
Flow diagram of the participants included in the study.

Out of the 434 individuals invited to participate, 370 individuals were assessed for eligibility. Among these, 130 were excluded, resulting in the final recruitment of 240 participants, and at the end, 215 were available for the final analysis after completing the full survey without missing data. Further information can be accessed in Fig. 1.

Among 215 participants in this study, the age of the participants ranged from 50 to 97 years with a mean age of 67.77 (11.77) years, a mean BMI was 27.01(5.48) kg/m^2^. The mean age for participants when they gave birth for the first time was 22.82 (3.96) years. Only 16.10% of the participants had a caesarean delivery. Moreover, individuals with a high risk of sarcopenia were 29.80%. Table 1 presents the demographic and reproductive characteristics of the 215 women enrolled in the study.

**Table 1:** Demographic and health status characteristics of the participants.

	Mean (SD) or *n* (%)
**Age (years)**	67.77(11.77)
**BMI (kg/m** ^**2**^ **)**	27.01 (5.48)
**Education**	
Primary school	77) 35.80%)
High school	67) 31.20%)
University	71) 33.00(%
**Marital status**	
Married	91 (42.50%)
Not married	124 (57.50%)
**History of incontinence in the family**	
Yes	53 (24.70%)
No	162 (75.30%)
**Episiotomy**	
Yes	125 (67.20%)
No	61 (32.80%)
**Hysterectomy**	
Yes	41 (19.10%)
No	174 (80.90%)
**Natural delivery**	
Yes	171 (91.90%)
No	15 (8.10%)
**Caesarean delivery**	
Yes	30 (16.10%)
No	156 (83.90%)
**Smoking**	
Yes	34 (15.80%)
No	181 (84.20%)
**Risk of sarcopenia**	
High risk	64 (29.80%)
Low risk	151 (70.20%)

### Univariate analysis

According to Mann-Whitney U test, incontinence symptoms were significantly different between participants who had a history of UI in the family and those who didn’t. Spearman correlation showed that age (*r*_*s*_=0.216, *p* < 0.01), SARC-F score (*r*_*s*_=0.460, *p* < 0.01), age of giving birth (*r*_*s*_ = − 0.209, *p* < 0.01) number of hospital visits (*r*_*s*_=0.185, *p* < 0.01), number of medications taken (*r*_*s*_=0.356, *p* < 0.01) number of diseases (*r*_*s*_=0.270, *p* < 0.01) and number of complications during birth (*r*_*s*_=0.180, *p* < 0.01) were significantly associated with incontinence scores Tables 2 and 3.

**Table 2:** Health and obstetric predictors of urinary incontinence.

Incontinence subscale	
**Variables tested**	***p*** **value**
Smoking	0.111
**Caesarean delivery**	0.868
**Natural delivery**	0.574
**History of incontinence in the family**	**0.003**
**Twins**	0.083
**Hysterectomy**	0.737
**Episiotomy**	0.767
**Giving birth or not**	0.481

**Table 3:** Correlation between predictors and urinary incontinence.

Incontinence subscale		
**Variables tested**	***r***_***s***_	***p*** **value**
BMI	0.091	0.186
**Age**	0.216	**0.001**
**Sarcopenia**	0.460	**< 0.001**
**Number of deliveries**	0.064	0.351
**Age when giving birth**	− 0.209	**0.004**
**Number of visits to hospital in the last year**	0.185	**0.006**
**Number of medications taken**	0.356	**< 0.001**
**Number of diseases**	0.270	**< 0.001**
**Number of complications during birth**	0.180	**0.008**

^1^Spearman correlation.

### Multivariate analysis

All of the significant predictors of the univariate analysis were entered into the multivariate linear regression model. Our multivariate regression model demonstrated that being at high risk of sarcopenia, age when giving first delivery, and number of complications during birth were all significant predictors of incontinence.

Among the significant predictors, high risk of sarcopenia and the number of complications during birth were positively associated with an increased risk of incontinence. However, age when giving birth for first time was negatively associated with the risk of incontinence. The R^2^ was 0.373, indicating that the included predictors in this model explained approximately 37.3% of the variance in the dependent outcome. Detailed results of the multivariate linear regression model are shown in Table 4.

**Table 4:** Multivariate linear regression analysis for urinary incontinence predictors.

Predictors	β	95%C (upper bound, lower bound)	*p*-value
**History of incontinence in the family**	0.128	− 0.203, 3.264	0.083
**Age**	− 0.006	− 0.077, 0.072	0.942
**Sarcopenia**	0.386	0.359, 0.994	**< 0.001**
**Age when giving first delivery**	− 0.157	− 0.413, − 0.017	**0.034**
**Number of visits to the hospital in the last year**	− 0.113	−1.526, 0.200	0.131
**Number of medications taken**	0.167	− 0.050, 1.329	0.069
**Number of diseases**	0.015	− 0.539, 0.650	0.854
**Number of complications during birth**	0.196	0.248, 1.955	**0.012**
**R** ^**2**^	0.373		
**Adjusted R** ^**2**^	0.335		
**F value**	9.827		

## Discussion

Our study suggested that 29.80% of the participants were at high risk of sarcopenia. Similarly, a previous study in Hungary with comparable participant characteristics, which investigated 100 women aged ≥ 50 years using SARC-F, reported a prevalence of 31%^18^.

Our results showed that only having a high risk of sarcopenia was a consistently significant risk factor of UI in univariate and multivariate analyses. One of the rare studies to investigate this association was a cross-sectional study conducted by Erdogan et al., suggesting a similar finding to our study, as sarcopenia was an independent risk factor for UI in their sample, which consisted of 802 females aged ≥ 60 years^19^.

Another prospective longitudinal cohort study by Parker-Autry et al. investigated 673 female participants who didn’t have UI symptoms at the beginning of the study and followed them for 4 years, they noticed that women who developed UI of any type had a critically worsening Short Physical Performance Battery test (SPPB) and especially in standing balance score, moreover, they observed deterioration of skeletal muscle mass and strength which are the main components of sarcopenia. Additionally, sarcopenia was more prevalent among participants with an incidence of UI compared with participants who stayed continent during the 4 years, suggesting that UI occurred simultaneously with sarcopenia^33^.

Previous studies suggested that stress urinary incontinence and urge incontinence are affected by pelvic floor muscle activation^22,34^. In addition, previous studies suggested that stress incontinence might be caused by a deficiency in the sphincter muscle that closes off the bladder channel. Regarding urgency incontinence pathogenesis, it includes pelvic floor muscle weakness besides abnormalities in bladder receptors and peripheral and central innervation deficits^34^. Due to all the previous, we can suspect that adequate abdominal and pelvic floor muscle strength is a major component in preventing both stress and urgency incontinence. In other words, sarcopenia might be related to both urge and stress incontinence as sarcopenia affects all skeletal muscles of the body including the pelvic floor and abdominal muscles.

Another possible interpretation for the association between sarcopenia and UI is the role of UI in aggravating the symptoms of sarcopenia by limiting PA level and encouraging a sedentary lifestyle, which might cause more deterioration of muscle mass and function^19^.

Previous studies examined components of sarcopenia but not the full definition of the disease showed inconsistent results. Park et al. investigated the association between muscle loss and UI; however, they didn’t find a significant association^34^. Another observational cohort study inspected the association between UI on one side and body composition and muscle strength on the other side for 3 3-year duration, suggesting that body composition and muscular strength are associated with SUI but not with UUI among older women^35^. As an increased risk of sarcopenia may contribute to the development of UI, its prevention or delay could be beneficial. Exercise plays a key role in this regard, resistance exercise in particular, as it enhances muscle strength, increases muscle mass, and improves muscle function^13,15,36^.

Unexpectedly, increased age at first delivery was a protective factor against UI this suggests that participants who gave birth for the first time at an older age had less risk of developing UI. This factor was not studied adequately in the literature. A possible explanation of these results might be that females who give birth at a younger age probably would have a higher number of deliveries in the future, which is a well-established risk factor for incontinence^37,38^. Further analysis in our study revealed a significant negative correlation between participants’ age and their age when giving birth for the first time (*r*_*s*_ = − 0.241, *p* < 0.01), which emphasises the relationship discussed above, in other words, older participants tend to have their first delivery at a younger age in our sample. This trend was also observed in other countries. For instance, a previous demographic study suggested that changes in educational enrolment explain a significant part of the increase in the age at first birth in Britain and France^39^. Nevertheless, the number of deliveries was not associated with UI in our study. These findings might be due to the small sample size and the huge variation among the different age groups, as our study included the youngest-old, middle-old, and oldest-old individuals^40^. Moreover, it’s worth mentioning that a large percentage of our participants have mild or non-UI symptoms, which might explain these results after considering the heterogeneity in our sample.

In this study, the number of diseases was associated with more severe UI symptoms; similar findings were suggested in a previous study^41^. Moreover, the age of the participant was correlated with UI score in the univariate analysis which was also similar to previous findings in the literature^37,38,42,43^ however, in the multivariate model it was not significant, this might be due to the fact of heterogeneity of our sample, which included middle- and old-aged women. Medication usage was also correlated with UI, similar results were found in a previous study in Turkey^44^. Regarding the history of incontinence in the family, it was also a significant factor in our study, this is in line with previous studies^37,44^ some of the interpretations that were suggested regarding this factor are the genetic and shared environmental factors^45^yet the genetics of UI is not clearly understandable^46^.

It’s also worth mentioning that type of delivery (caesarean vs. vaginal) was not a considerable factor in our study, yet Sarici et al. study they suggested that women with vaginal delivery had more severe symptoms of urinary incontinence^38^. Additionally, another study revealed that caesarean section delivery was a protective factor against UI^47^. It has been reported that vaginal deliveries could cause anatomic and functional changes in the pelvic floor, consequently, leading to hypermobility of the bladder base and increasing the risk of stress urinary incontinence^48^. The reason behind the contradiction in our findings compared to previous investigations might be due to the noticeably small number of participants who had a caesarean delivery (30 participants) out of the total number of participants who gave birth (186 participants).

In our study, the number of complications during delivery significantly predicted increased risk of UI in both univariate and multivariate analysis, previous meta-analysis has shown that childbirth complications (e.g. perineal tear) are risk factors for UI^49^.

BMI was not associated with UI in our study, however, previous investigations have shown opposite results^38,43^furthermore, it has been suggested that increased BMI is associated with higher intraabdominal pressure, which may, over time, lead to neuropathy and weakening of the pelvic floor muscles. Nevertheless, further neurophysiological studies are still needed to understand the pathophysiology behind this association^50^. Additionally, Metabolic Syndrome—a complex disorder comprising numerous interrelated pathophysiologic entities, including obesity, hypertension, dyslipidemia, hyperglycemia/insulin resistance—has also been proposed as a contributing factor. Previous findings suggest that Metabolic Syndrome components might be independently associated with both stress and urgency urinary incontinence^51^.

However, another investigation revealed that a higher BMI is correlated with increased pelvic floor muscle strength (PFMS)^52^. A cross-sectional study investigating associated factors with sarcopenia among 104 nursing home residents suggested that obesity might act as a protective factor against sarcopenia^53^subsequently, it might reduce the risk of UI.

Our study has the following strengths: to the best of our knowledge few studies have investigated the association between the risk of sarcopenia and UI. Furthermore, our study used standardised and validated questionnaires which are specially designed to measure incontinence and risk of sarcopenia, this probably enhanced the accuracy of our results, and we encourage the use of such validated screening tools, as this might enhance the development of healthcare strategies regarding this issue. Moreover, to achieve accuracy with answers and obtain a high response rate the survey was filled with the presence of interviewers.

However, our study has the following limitations, although SARC-F is a reliable screening tool for assessing the risk of sarcopenia, it is not sufficient for a conclusive diagnosis of sarcopenia. Moreover, we might have a risk of response bias as there might be differences in characteristics between those who respond and those who refuse to participate in the study, which might affect the estimates of associations and the internal validity of our study. In addition, the results of this study should be interpreted carefully as it is not reliable to build concrete conclusions or causality relationships based on observational investigations. Additionally, even though the participants and data collectors were blinded to the specific hypothesis of the study, interviewer bias still may have occurred. Moreover, we didn’t observe our participants for long periods to observe whether UI symptoms worsened with the occurrence of sarcopenia or vice versa. Finally, most of the risk factors investigated in this study showed a weak association with UI symptoms, only sarcopenia was an exception.

This study highlights the importance of screening for sarcopenia to early identify possible risks and utilise appropriate interventions that aim to prevent or decrease the risk of developing UI. Further longitudinal studies with large sample sizes investigating the association between sarcopenia and UI while controlling for other confounding factors are recommended to reach more robust conclusions.

## Conclusion

This study suggested that being at high risk of sarcopenia is a risk factor for UI. Additionally, age when giving the first delivery and the number of complications during birth were risk factors for UI. Further longitudinal studies are recommended to investigate risk factors of UI over extended periods while controlling for confounding factors.

## Data Availability

The datasets used during the current study available from the corresponding author on reasonable request.

## Abbreviations

BFLUTS: Bristol female lower urinary tract symptoms
BMI: body mass index
EWGSOP: European working group on sarcopenia in older people
ICIQ-FLUTS: international consultation on incontinence questionnaire-female lower urinary tract symptoms modules
LUTS: lower urinary tract symptoms
PFMS: pelvic floor muscle strength
Rs: Spearman’s rank correlation coefficient
SD: standard deviation
STROBE: strengthening the reporting of observational studies in epidemiology
SUI: stress urinary incontinence
UI: urinary incontinence
UUI: urge urinary incontinence
